# Search engines and short video apps as sources of information on acute pancreatitis in China: quality assessment and content assessment

**DOI:** 10.3389/fpubh.2025.1578076

**Published:** 2025-06-04

**Authors:** Shishuang Jiang, Youlian Zhou, Jun Qiu, Xiaohong Gou

**Affiliations:** ^1^Intensive Care Unit, The Affiliated Yongchuan Hospital of Chongqing Medical University, Chongqing, China; ^2^Intensive Care Unit, Chengdu Integrated TCM and Western Medicine Hospital, Chengdu, China

**Keywords:** acute pancreatitis, web pages, TikTok, BiliBili, quality

## Abstract

**Background:**

Acute pancreatitis is a prevalent condition in China. A plethora of information pertaining to acute pancreatitis is readily available on the Internet, including on major search engines and various short video applications. The objective of this study was to evaluate and compare the quality, content completeness, and accuracy of information related to acute pancreatitis on web pages of the four major search engines and short videos on the two major short video platforms.

**Materials and methods:**

A search was conducted on four major search engines (Bing, Baidu, Haosou, and Sougou) and two short video apps (TikTok and BiliBili) using the Chinese keyword “acute pancreatitis.” The sources can be divided into four categories: scientific resources, news/media reports, industrial/commercial profit organizations, and healthcare providers. The quality of the web pages and short videos was evaluated using the DISCERN instrument, the Global Quality Score (GQS), and the Journal of the American Medical Association (JAMA). In accordance with established guidelines and reviews of acute pancreatitis, two lists and scoring systems were devised for the evaluation of content comprehensiveness and accuracy.

**Results:**

A total of 120 unique web pages and 120 unique videos were identified using four search engines and two short video applications, respectively. The most prevalent identity among those producing short videos was that of healthcare providers. With regard to the source of the web pages, the most prevalent category was that of industrial/commercial profit organizations. The median DISCERN total score, median GQS score, and median JAMA score were 26, 3, and 2, respectively. Web pages exhibited significantly higher ratings than short videos (*p* < 0.001, *p* = 0.003, and *p* < 0.001). The median overall content score for the web page was 8 (interquartile range [IQR] 4–13), and the median guideline-related score was 2 (IQR 0–4), indicating that the web page’s content was deficient in terms of completeness and accuracy. In comparison, the performance of the short video was found to be even more deficient.

**Conclusion:**

In China, most web pages pertaining to acute pancreatitis were produced by industrial/commercial profit organizations, but the quality of the information provided by these entities was found to be the lowest. The majority of short videos were created by healthcare providers, but the overall quality of these videos was found to be inadequate. In general, the quality of both web pages and short videos is suboptimal. Nevertheless, the quality of web pages was found to be superior to that of short videos. In terms of completeness and accuracy, both the web page and the video exhibit significant deficiencies that are cause for concern.

## Introduction

Acute pancreatitis (AP) represents a common inflammatory disease affecting the exocrine pancreas. The condition is typified by acute abdominal discomfort and multi-organ dysfunction, with the potential for progression to pancreatic necrosis and sustained organ failure. The estimated incidence of acute pancreatitis is between 110 and 140 cases per 100,000 population, with an estimated 300,000 or more emergency department visits per year in the United States (1, 2). Approximately 80% of patients present a mild-to-moderate form of the disease (absence of organ failure >48 h). Nevertheless, one in five patients develop a severe disease form with a mortality rate of approximately 20% (3–5). A notable rise in the prevalence of AP was documented, with an increase from 9.48 cases per 1,000 hospitalizations in 2002 to 12.19 cases per 1,000 hospitalizations in 2013 (6). Gallstones and alcohol abuse represent the two most common etiologies of acute pancreatitis (AP), collectively accounting for 80% of cases (7). Severe hypertriglyceridemia (HTG) is a well-established and the most common cause of acute pancreatitis (AP) subsequent to alcohol and gallstone disease. It is estimated that this condition may be responsible for up to 10% of all cases of pancreatitis (8). It has been demonstrated that modifying lifestyle behaviors in individuals at risk can reduce the incidence of gallstones (9). Additionally, avoiding alcohol and achieving a healthy weight can prevent the other two causes of the disease. It is therefore evident that educational initiatives and modifications to lifestyle behaviors represent crucial elements in the prevention of acute pancreatitis.

As the influence of Internet technology has grown, the use of electronic information has become the dominant form of information, superseding the use of paper-based information. Individuals have increasingly relied on the Internet as a primary source of health information. The World Wide Web provides a novel avenue for individuals and their relatives to obtain health-related information. In 2014, 84% of the United States population were internet users, with 72% of this demographic searching for health-related information online (10). In contrast to traditional textual information, which is often perceived as requiring a significant investment of time to read, videos are becoming an increasingly popular medium due to their capacity to engage and inform in a more visually appealing manner (11). The video-sharing platforms YouTube, TikTok, and BiliBili are widely utilized, with an international user base. Despite its status as the most extensive global platform for long-form video content, YouTube remains inaccessible in China (12). TikTok has been downloaded more than 200 million times in the United States alone, is available in over 150 countries, and has a user base exceeding one billion (13). BiliBili has amassed a considerable user base, numbering in the millions, which can be attributed to the convenience, interactivity, and diversity offered by the platform (14). The dissemination of high-quality health information can facilitate the process of health education and enhance the health literacy of the general public. Conversely, the circulation of low-quality health information has the potential to mislead the general public, which could ultimately result in significant adverse outcomes. In 2016, Fisher et al. conducted an analysis of the quality of web pages about idiopathic pulmonary fibrosis on Google, Yahoo, and Bing. The researchers’ findings revealed that the online information available was frequently lacking in completeness, accuracy, or timeliness (15). Moreover, recent studies have indicated that the quality of videos on *Helicobacter pylori*, gallstone disease, and liver cancer on both TikTok and BiliBili is suboptimal (13, 14, 16).

In 2013, the American College of Gastroenterology (ACG) published clinical guidelines for the diagnosis and management of acute pancreatitis (17). Subsequently, in 2018, these were updated with a particular focus on the early management aspects of acute pancreatitis (18). Furthermore, a literature review on the subject of acute pancreatitis was published in 2022 by Michael et al. (19). Despite the existence of comprehensive, widely read and highly cited guidelines and reviews on the subject, non-professionals are often misleadingly influenced by incorrect or outdated information obtained from online sources related to the condition.

The objective of this study was to assess the overall quality of information about acute pancreatitis available online, including web pages and video platforms. In addition, an assessment of the completeness and accuracy of the content was conducted through comparison with the established guidelines and reviews.

## Methods

### Ethical considerations

It should be noted that no clinical data, human specimens, or laboratory animals were utilized during this study. All data were obtained from publicly accessible sources, namely web pages and short video applications. Given that the data did not contain any personally identifiable information, it was determined that an ethical review was not a prerequisite.

### Search strategy

To generate a list of web pages or videos comparable to those generated by an individual with limited medical, internet, or computer expertise, we employed a straightforward search method. The search was conducted using the keyword“急性胰腺炎” (acute pancreatitis). All searches were conducted on October 10, 2024. In order to reduce the influence of ‘social media algorithms’ which influence user content, a new TikTok account and a new BiliBili account were created, and the Chinese version of TikTok was utilized. The present study was limited to the top 60 videos, as prior research has demonstrated that over 95% of users only view the initial 60 videos (14). In accordance with the engine rankings, from January to August 2024, the most prevalent Chinese search engines in China were Bing, Baidu, Haosou, and Sougou (20). In order to reduce the influence of prior queries, the location services were disabled and all cookies, previous search history, and temporal internet files were eliminated before and between searches. A search was conducted using the aforementioned keyword in Bing, Baidu, Haosou, and Sougou. Prior academic research has demonstrated that the typical individual will seldom peruse more than 30 websites in the process of conducting online searches (21). Accordingly, the initial 30 links returned by each search engine were subjected to analysis.

The following exclusion criteria were employed: duplication; personal experience or experience; discussion groups or open forums; in non-Chinese languages; direct access is prohibited, and access is required using a password, registration, or payment of a fee; directed toward a professional audience; and any website that serves primarily as a portal to another website. Additionally, web pages that are exclusively video or audio are not included in this analysis.

### Source classification

The sources of the web pages and videos were classified into the following categories: scientific resources (e.g., academic institutions and government organizations), news/media reports, industrial/commercial profit organizations, and healthcare providers (e.g., hospitals or doctors).

### Quality assessment

To assess the quality of the information presented in web pages and videos, the DISCERN instrument, the Journal of the American Medical Association (JAMA), and the Global Quality Score (GQS) were employed.

The DISCERN tool is a standardized, validated instrument comprising 16 items that has been designed to assist patients and information providers in appraising the reliability and quality of written health information and treatment choices (22). Furthermore, the DISCERN tool has been tested for use in assessing health information available on the Internet (22, 23). The initial 15 questions are closed, and respondents are required to indicate their level of agreement or disagreement with each statement on a scale of 1–5, where 1 signifies a definitive negative response and 5 represents a definitive positive response. The final item assesses the overall quality of the presented information on a scale of 1 to 5, with 1 indicating low quality and 5 indicating high quality (Supplementary Table 1). The tool is comprised of three sections. The objective of Section 1 (Questions 1 to 8) is to evaluate the reliability of the publication. Section 2 (Questions 9 to 15) is concerned with the quality of the information provided regarding the treatment. Ultimately, Section 3 (Question 16) assesses the overall quality of the publication. The overall DISCERN scores ranged from 16 to 80 and were then categorized according to the following standards: very poor (16–26), poor (27–38), fair (39–50), good (51–62), and excellent (63–80) (24, 25). The JAMA are designed to assess the following core standards: authorship, attribution, disclosure, and currency (Supplementary Table 2). Each item is assigned a value of 0 or 1, where 0 indicates the absence of the specified characteristic and 1 indicates its presence. Moreover, the Global Quality Scale (GQS) was utilized to assess the quality of the educational content, with specific consideration given to the evaluation of quality, the comprehensiveness of the listed information, and its utility for patients (Supplementary Table 3) (26–29). The GQS scores are expressed on a scale of 1 to 5, with a maximum score of 5 representing the highest quality.

### Completeness and accuracy assessment

To evaluate the completeness of the information provided regarding acute pancreatitis on the web pages and videos, a checklist was utilized that was specifically developed for this study and based on reputable and internationally respected sources (19, 30). The checklist comprises seven sections. The following seven sections were considered: (1) etiology, (2) clinical presentation, (3) diagnostic criteria, (4) disease severity, (5) risk stratification, (6) management, and (7) relapse prevention. A total of 22 pre-defined terms are associated with the seven sections of the disease, with each term being worth one point. Subsequently, the web page or video was evaluated on a scale of 0 to 22, with 22 signifying the highest level of comprehensiveness (see Supplementary Table 4). To assess the accuracy of the information provided on the web pages and videos, the recommendations set forth in the 2013 American College of Gastroenterology guideline and the 2018 American Gastroenterological Association Institute guideline were utilized (17, 18). All recommendations were extracted independently by two authors, and any discrepancies were resolved through discussion. After a period of deliberation among all participants, including leading experts in critical care medicine, it was decided to retain the strong recommendations and then select only those that were essential to decision-making. Ultimately, the study incorporated 15 recommendations, as detailed in Supplementary Table 5. The evaluation of accuracy was conducted in accordance with a standardized framework based on selected guideline recommendations. This methodology has been previously employed in similar studies within this field (15, 31). Each recommendation was scored according to the following criteria: 0 (if absent or described incorrectly), 1 (present and described incompletely), or 2 (present and described completely).

The scores were evaluated by two physicians from the Intensive Care Unit, Jun Qiu and Youlian Zhou, in accordance with the specified criteria. The arbitrator (Shishuang Jiang and Xiaohong Gou) resolved the inconsistencies in viewer scores and provided the definitive results. Subsequently, a consensus was reached among all authors with regard to the ratings.

### Statistical analyses

The data are not normally distributed. For descriptive statistics, the median (interquartile range) was used. The Mann–Whitney U test was used to compare two groups of quantitative data. In addition, the Kruskal-Wallis test was used to compare multiple sets of quantitative data, while Dunn’s multiple comparison test was used to compare between groups. A *p*-value of less than 0.05 was considered statistically significant. All data were analyzed using the Statistical Package for the Social Sciences (SPSS) version 27 software.

## Results

### Selection process

**Figure 1:**
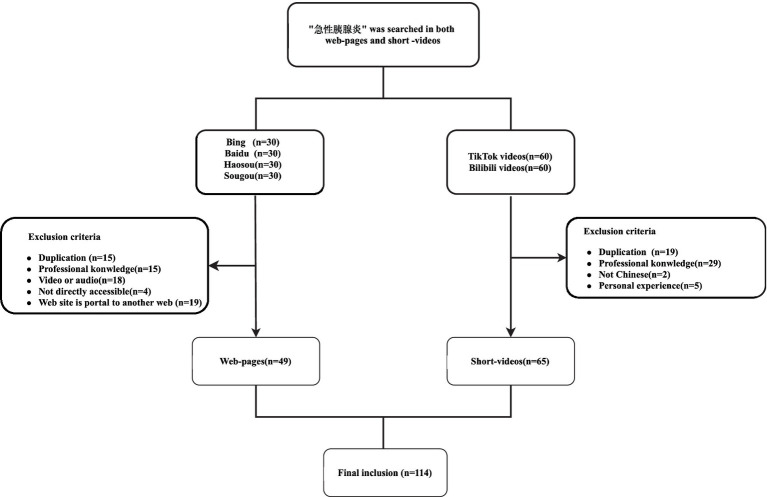
Videos and web-pages screening flowchart.

A total of 120 unique web pages and 120 unique videos were identified using four search engines and two short video applications, respectively. As illustrated in Figure 1, the inclusion criteria were met by 49 websites and 65 videos.

### Sources for web pages and short videos

**Figure 2:**
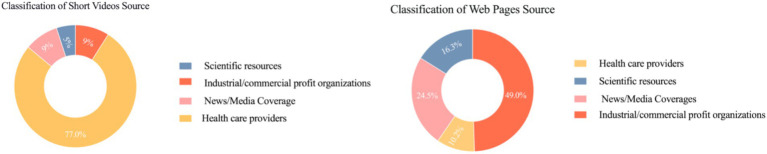
Classification of source.

The most prevalent identity among short video producers was that of healthcare providers (*n* = 50, 77.0%), followed by news/media reports (*n* = 6, 9.0%) and industrial/commercial profit organizations (*n* = 6, 9.0%), with scientific resources representing the smallest proportion (*n* = 3, 5.0%). Furthermore, regarding the source of the web pages, the most prevalent category was that of industrial/commercial profit organizations (*n* = 24, 49%), of which 87.5% were advertisements for clinics and private hospitals and 12.5% were scientific articles, followed by news/media reports (*n* = 12, 24.5%), scientific resources (*n* = 8, 16.3%), and healthcare providers (*n* = 5, 10.2%) (Figure 2).

### Web pages vs short videos

The median DISCERN total score, median GQS score, and median JAMA score were 26, 3, and 2, respectively (Table 1), with web pages exhibiting significantly higher ratings than short videos (*p* < 0.001, *p* = 0.003, and *p* < 0.001). The median overall content score for the web page was 8 (interquartile range [IQR] 4–13), and the median guideline-related score was 2 (IQR 0–4), indicating that the web page’s content was deficient in terms of completeness and accuracy. In comparison, the performance of the short video was found to be even more deficient.

**Table 1:** Scores of webpages and short video.

Variable	Total score, median [IQR]	Web-pages (*n* = 49), median [IQR]	Short-videos (*n* = 65), median [IQR]	*p*
DISCERN score	26 (23, 36)	37 (27.5, 54)	24 (22.5, 26.5)	0.000
Reliability (Questions 1–8)	15 (14, 20)	20 (16, 30)	14 (14, 15)	0.000
Treatment choices (Questions 9–15)	7 (7, 15)	16 (7, 21)	7 (7, 7)	0.000
Overall information quality (Question 16)	3 (2, 3)	3 (2, 4)	2 (2, 3)	0.002
Global score	3 (2, 3)	3 (2, 4)	2 (2, 3)	0.003
JAMA	2 (2, 3)	3 (2, 4)	2 (2, 2)	0.000
Content total score	4 (1, 9)	8 (4, 13.5)	3 (0.5, 5.5)	0.000
Guideline score	0 (0, 2)	2 (0, 4.5)	0 (0, 0)	0.000

### Characteristics of the short videos

Table 2 presents the descriptive statistics of the videos from disparate sources. The videos have received a total of 104 to 1,734 likes, with a median value of 362. Additionally, the videos received 6–187 comments, with a median value of 33; and 22–464 saves, with a median value of 85. Moreover, the median video duration was 92 s. The median duration was 82 s for healthcare providers and 73 s for news/media reports, which were both significantly shorter than the median durations observed for scientific resources and industrial/commercial profit organizations. The median number of days since publication for industrial/commercial profit organizations was 461, while the figure for healthcare providers was 527. These figures were markedly shorter than those observed for scientific resources and news/media reports.

**Table 2:** Analysis of short-video characteristics.

Source type	Likes, median [IQR]	Comments, median [IQR]	Saves, median [IQR]	Duration time (seconds), median [IQR]	Days since published, median [IQR]
Total	362 (104, 1734)	33 (6, 187)	85 (22, 464)	92 (52, 143)	554 (479, 850)
Scientific resources*	710 (4, -)	33 (0, -)	348 (11, -)	294 (84, -)	1993 (339, -)
News/media coverage	1, 353 (318, 7, 212)	35 (12, 300)	308 (120, 1, 153)	73 (38, 132)	956 (555, 962)
Industry/profit organization	371 (18, 1766)	24 (0, 190)	138 (42, 722)	255 (123, 456)	461 (249, 731)
Healthcare providers	250 (107, 1760)	31 (9, 199)	71 (20, 364)	82 (49, 139)	527 (496, 774)

*Quantile distance could not be calculated due to insufficient data.

### Comparison of video scores from different sources

The median DISCERN total scores were found to be significantly higher for industrial/commercial profit organizations than for news/media reports and healthcare providers (*p* < 0.05). The individual reliability score (questions 1–8) was found to be significantly higher for industrial/commercial profit organizations than for news/media reports (*p* < 0.05). The overall quality of industrial/commercial profit organizations was found to be superior to that of healthcare providers (*p* < 0.05), as evidenced by the results of Question 16. The JAMA Benchmark scores for videos from a variety of sources exhibited no discernible discrepancies. The median GQS scores for news/media reports and healthcare providers were found to be significantly lower than those for industrial/commercial profit organizations (*p* < 0.05). In general, the content total scores (total score of 22) and the guideline-related scores (total score of 30) were found to be inadequate. With regard to content scores, the proportion of short videos achieving a score of 0 in each of the seven areas of etiology, clinical presentation, diagnostic criteria, disease severity, risk stratification, management, and relapse prevention was significantly higher than the proportion achieving a score of 1, particularly in the areas of diagnostic criteria, risk stratification, and management (see Supplementary Table 6). In the context of the guide-related questions, a single question was identified in which a short video received a score of 2. Remaining 14 questions did not include a short video that attained a score of 2. Moreover, for each question related to the guides, the proportion of short videos receiving a score of 0 exceeds 90%, reaching a maximum of 98.46%. The industrial/commercial profit organizations exhibited the highest content total scores, yet the median score was only 6. About guideline-related scores, the same organizations also demonstrated the highest score, but the median score was only 1 (Table 3).

**Table 3:** Short-videos rank sum test.

Variable	Scientific resources (*n* = 3)	News/Media coverage (*n* = 6)	Industry/Profit organization (*n* = 6)	Healthcare providers (*n* = 50)	*p*
Total DISCERN score	24 (24, -)	21.5 (20, 24.8)	30 (27.8, 41)	23 (22.8, 24)	0.003^a^
Reliability (questions 1–8)	14 (14, -)	12.5 (11, 14)	19.5 (14, 27.8)	14 (14, 15)	0.005^b^
Treatment choices (questions 9–15)	7 (7, 7)	7 (7, 8)	7 (7, 13.8)	7 (7, 7)	0.477
Overall quality (question 16)	3 (3, 3)	2 (2, 2.3)	3.5 (2.8, 4)	2 (2, 3)	0.007^c^
JAMA	2 (2, -)	2 (1.8, 2.5)	2 (2, 2)	2 (2, 2)	0.770
GQS	3 (3, 3)	2 (1.8, 2.3)	3.5 (2.8, 4)	2 (2, 3)	0.002^a^
Content	5 (2, -)	3.5 (2.5, 7.5)	6 (2.5, 10.3)	3 (0, 4)	0.070
Guideline scoring	0 (0, 0)	0 (0, 0.5)	1 (0, 2)	0 (0, 0)	0.247

Data are presented as median, quartiles. Superscript letters indicate: ^a^ News/Media coveragevs Industry/Profit organization and Healthcare Providers vs industry organization. ^b^ News/Media Coverage vs Industry/Profit Organization. ^c^ Healthcare Providers Vs Industry/Profit Organization. All comparisons were subjected to Bonferroni correction.

### Comparison of web pages scores from different sources

The median total DISCERN score for industrial/commercial profit organizations was found to be significantly lower than that for science resources and news/media reports (*p* < 0.05). In Section 1, the DISCERN scores for industrial/commercial profit organizations exhibited a markedly inferior performance in comparison to the other three sources (*p* < 0.05). This indicates that the reliability of web pages belonging to industrial/commercial profit organizations may be inferior to that of other sources. In Section 2, a statistically significant difference was observed in the DISCERN scores of industrial/commercial profit organizations and those of scientific resources (*p* < 0.05). The findings suggest that web pages from industrial/commercial profit organizations are less satisfactory in providing treatment information than web pages from scientific resources. In Section 3, a statistically significant decline was observed in the DISCERN score for industrial/commercial profit organizations when compared with the other three sources (*p* < 0.05). In general, the quality of web pages from industrial/commercial profit organizations was found to be inferior to that of other sources. No discernible discrepancies were identified in the JAMA Benchmark scores for web pages from disparate sources. The median GQS scores were found to be significantly lower in industrial/commercial profit organizations when compared to those in news/media reports and scientific resources, as well as in healthcare providers (*p* < 0.05). It was found that the content total scores and the guideline-related scores for web pages were generally unsatisfactory. With respect to etiology, the proportion of pages that received a score of one was greater than the proportion of pages that received a score of zero in instances of gallstones, alcohol, and triglyceridemia. However, for medical causes, hypercalcemia and infections, the proportion of pages that received a score of zero was greater than the proportion of pages that received a score of one. In clinical manifestation, the proportion of pages scoring 1 was higher than the proportion of pages scoring 0. However, the proportion of pages scoring 0 was significantly higher than the proportion of pages scoring 1 in the remaining five domains, especially in risk stratification. As demonstrated in Supplementary Table 7. The percentage of web pages achieving a score of zero was found to be the highest among all guide-related questions, with a range from a low of 63.27% to a high of 95.92%. Conversely, the percentage of pages achieving a score of two was found to be the lowest, with certain questions exhibiting a percentage of zero. The highest content score was observed for news and media reports, which achieved a score of 14 out of 22, followed by scientific resources, which scored 13. The lowest score was observed for industrial/commercial profit organizations, which achieved a score of 4.5. A statistically significant discrepancy was observed between the latter and the first two. Regarding the scores related to the guidelines, all sources demonstrated a low level of performance, with industrial/commercial profit organizations exhibiting a score of zero (see Table 4).

**Table 4:** Web-pages rank sum test.

Variable	Scientific resources (*N* = 8)	News/Media coverage (*N* = 12)	Industry/Profit organization (*N* = 24)	Healthcare providers (*N* = 5)	*p*
Total DISCERN score	47.5 (39.3, 60.5)	54 (37.8, 60.8)	27.5 (23.3, 36.8)	48 (37.5, 61.5)	0.000^a^
Reliability (questions 1–8)	26 (17, 34.5)	30 (22.5, 35.2)	16 (14, 19)	25 (23, 33.5)	0.000^b^
Treatment choices (questions 9–15)	19.5 (18.3, 22)	19 (14.5, 21)	9 (7, 16.8)	21 (10.5, 24)	0.009^c^
Overall quality (question 16)	3.5 (3, 4)	4 (3, 4)	2 (2, 3)	4 (3, 4)	0.000^b^
JAMA	3 (0.3, 3)	2.5 (2, 3)	4 (2.3, 4)	3 (2, 3)	0.077
GQS	3 0.5 (3, 4)	4 (3, 4)	2 (2, 3)	4 (3, 4)	0.000^b^
Content	13 (12, 14)	14 (6, 17.5)	4.5 (3, 6)	11 (7.5, 19)	0.001^d^
Guideline scoring	3.5 (2, 8)	4 (0.5, 8)	0 (0, 1.8)	3 (0, 5.5)	0.002^d^

Data are presented as median, quartiles. Superscript letters indicate: ^a^ Industrial/commercial profit organizations vs Scientific Resources and Industrial/commercial profit organizations vs News/Media reports. ^b^ Industrial/commercial profit organizations vs Scientific Resources and Industrial/commercial profit organizations vs Healthcare Providers, Industrial/commercial profit organizations vs News/Media reports. ^c^ Industrial/commercial profit organizations vs Scientific Resources. ^d^ Industrial/commercial profit organizations vs News/Media reports and Industrial/commercial profit organizations vs Scientific Resources. All comparisons were subjected to Bonferroni correction.

## Discussion

To the best of our knowledge, this is the original study to compare the quality of web pages about acute pancreatitis on China’s most popular search engine with the quality of videos about acute pancreatitis on two of China’s most popular video apps. It is our hope that this study will provide valuable insight that can be utilized to enhance public awareness of acute pancreatitis.

Chang-Li and colleagues conducted an analysis of the global burden of acute pancreatitis in 204 countries and territories. The findings indicated a 62.9% increase in the number of acute pancreatitis cases, from 1,727,789.3 in 1990 to 2,814,972.3 in 2019. In 2019, the countries with the highest incidence of acute pancreatitis were India, followed by China and the United States. In 2019, the countries with the highest number of fatalities were India, Russia, and China (32). To prevent pancreatitis, Maxim S. Petrov et al. put forth the Holistic Prevention of Pancreatic Inflammation (HPP) framework. The framework identifies three levels of prevention, each targeting a specific population. The first level, primary prevention, targets the general population. The second level, secondary prevention, targets patients in the early stages of acute pancreatitis and chronic pancreatitis. The third level, tertiary prevention, targets patients with all forms of pancreatic inflammation at risk of complications (33). The study by Ammar Alsamarrai et al. contributed to primary prevention by demonstrating that cessation of smoking, control of body weight, limitation of alcohol intake, and increase in fruit and vegetable consumption are effective methods for the reduction of the risk of pancreatic disease (34). The primary objective of tertiary prevention is to prevent the various sequelae of pancreatitis. The Prediabetes Self-Assessment Screening Score After Acute Pancreatitis (PERSEUS) is the inaugural screening instrument designed to identify patients who have experienced an acute pancreatitis episode and are at an elevated risk of developing prediabetes (35). All variables included in the score are accessible to individuals and do not necessitate laboratory testing. Two variables, namely tobacco smoking and abdominal obesity, represent potentially modifiable risk factors that could be targeted with the objective of reducing the incidence of post-pancreatitis diabetes mellitus (PPDM). These findings indicate that acute pancreatitis represents a significant public health concern in China, with high morbidity and mortality rates. Engagement of the general population and patients through the HPP framework may prove an effective strategy for reducing the morbidity and post-pancreatitis sequelae associated with this condition.

As indicated in the 50th Statistical Report on China’s Internet Development Status, published by the China Internet Network Information Center, the nation’s Internet penetration rate reached 74.45% by June 2022, with 1.051 billion Internet users (36). A growing number of individuals in China are expressing concerns about their health and frequently seeking health-related information online prior to consulting with a medical professional. Nevertheless, a review conducted by the US Office of Disease Prevention and Health Promotion (ODPHP) has determined that the potential for harm from inaccurate online information is significant. The potential for harm can manifest in three distinct forms: (1) physical, from the administration of inappropriate treatments, adverse effects, or the neglect of disease; (2) emotional, from the experience of anxiety or the nurturing of false hope because of inaccurate diagnostic, prognostic, or therapeutic information; and (3) financial, from the expenditure of resources on ineffective health services or products (37). In a recent study, Tianyang Mao and colleagues conducted an evaluation of the quality of videos on the social media platform TikTok pertaining to the topic of acute pancreatitis. The findings indicated that the overall quality of the videos was inadequate (38). A review of the existing literature reveals a dearth of studies that assess the quality of web pages about acute pancreatitis. To further enhance our comprehension of the quality of information regarding acute pancreatitis on the Internet, we conducted this study.

In this study, an evaluation was conducted on the quality of 114 web pages and short videos pertaining to the topic of acute pancreatitis. In general, the quality of web pages and short videos on acute pancreatitis is found to be inadequate. Notwithstanding, the quality of web pages is markedly superior to that of short videos. Most of the web pages have been created by industrial/commercial profit organizations. Nevertheless, these organizations tend to receive relatively low scores in quality assessments. Similarly, prior research has demonstrated that most web pages addressing sleep apnea are commercial in nature. The information provided by these commercial sources was typically of a lower quality and more unreliable than that of non-commercial sources (39). This finding aligns with the results of our research. Conversely, most video producers were healthcare providers. The findings of the study indicate that healthcare providers demonstrated suboptimal performance about the quality assessment, which may be associated with the relatively shorter duration of the videos they posted. As previous studies have demonstrated, high-quality videos typically have longer durations than low-quality videos (40). Similarly, prior research has established that most video producers on both TikTok and BiliBili are health professionals (13, 16). Furthermore, both the Global Quality Score (GQS) and DISCERN scores indicated that the overall quality of short videos on both platforms is inadequate (14). These findings are consistent with our research.

The present study found that the percentage of short videos mentioning etiology, clinical presentation, diagnostic criteria, disease severity, risk stratification, management, and relapse prevention was very small, especially in the three areas of diagnostic criteria, risk stratification, and management. With regard to web pages, a significant proportion mentioned gallstones, alcohol, hypertriglyceridemia, and clinical manifestations. However, a considerable number did not refer to other aspects, particularly in the context of risk stratification. This phenomenon may exert a deleterious effect on public health perception and disease management. Firstly, the absence of information regarding diagnostic criteria and risk stratification has the potential to result in erroneous public perceptions of disease severity, delays in seeking medical treatment, or excessive panic. Secondly, the paucity of discussion regarding management strategies has the potential to compromise patient adherence and impact outcomes. Furthermore, although the webpage mentioned the common causes of the disease at a high rate, the one-sided emphasis on a single factor may mislead the public to ignore other risk factors (e.g., infections), which is not conducive to comprehensive prevention. A similar phenomenon has been observed in online resources for other diseases. In a recent study, Fisher and colleagues evaluated the content profile of web pages about idiopathic pulmonary fibrosis on the three primary search engines. The results demonstrated a dearth of comprehensive coverage of essential elements, including established diagnostic techniques, management strategies, and prognostic details (15). Moreover, the study established that web pages and short videos had significantly higher rates of failure to mention content relating to post-admission protocols that had been recommended by guidelines. In some cases, incorrect descriptions were also identified, for example, one web page clearly states the use of antibiotics to prevent infection in the treatment plan, but the guidelines explicitly state that prophylactic antibiotics are not routinely recommended. Moreover, about fluid resuscitation, the amount required was reported to be 3,500–4,000 mL/day by one web page. This statement was also found to be incongruent with the resuscitation time and the amount of fluid rehydration stipulated in the guidelines. This deficiency has the potential to exert a considerable negative effect on the accuracy and thoroughness of public health information that is accessible to the public. Firstly, patients may receive non-standardized treatment recommendations, increasing the likelihood of misdiagnosis, over-treatment, or under-treatment. Secondly, the lack of information may weaken the public’s scientific knowledge of disease management, such as neglecting relapse prevention measures, which may affect long-term prognosis. A previous study evaluated the accuracy of web pages on endometriosis in accordance with pertinent guideline recommendations. The findings indicated that the accuracy of these web pages was suboptimal (31). It is evident that online resources on this topic are severely lacking in terms of completeness and accuracy. It is unwise for the public to seek information about acute pancreatitis online, given the unreliability and potential risks associated with data of inadequate quality, completeness, and accuracy.

This study found no significant differences in JAMA scores for short videos and web pages from different sources. This corroborates the difficulty of using this tool to comprehensively assess information quality, due to its limited dimensionality (only four items). In contrast, the DISCERN and GQS scores revealed significant quality heterogeneity. The strength of the DISCERN scale lies in its systematic and comprehensive assessment by means of 16 questions, in particular the rigorous examination of the completeness of the information on treatment options. Conversely, GQS scores offer an assessment that is more closely aligned with patient experience in terms of overall quality and fluency. Nevertheless, the DISCERN scale does not encompass an evaluation of health literacy appropriateness (e.g., verbal readability, visual aids), which is a pivotal component of short-form video dissemination. The wide scope of the GQS may result in the generation of false positives, which could subsequently lead to the overestimation of well-produced, yet superficial video content. It is imperative that future tools address the disadvantages of both methods, incorporating health literacy and automated analyses (e.g., using natural language processing to detect completeness and accuracy of key messages) to more effectively assess the clinical value of short videos.

It should be noted that this study is subject to certain limitations. It is important to note that the search was conducted on a single computer in the Chengdu area of China. Consequently, it is not possible to discount the possibility of regional variations in the search, and thus the degree of variability, and therefore the generalizability, was not assessed in this study. Secondly, the present study included only web pages and videos in Chinese sourced from four search engines and two video-sharing platforms. Therefore, the findings may not be generalizable to other search engines and video platforms. Furthermore, the relatively modest sample sizes of some subgroups may potentially introduce errors, which could be mitigated by either increasing the sample sizes or expanding the search terms. Finally, as demonstrated in previous studies (41, 42), health information that is accessible via search engines frequently exceeds the recommended reading level of the general population. Consequently, it is imperative to evaluate the readability and comprehensibility of web pages and videos. However, a review of the extant Chinese literature failed to identify any validated tools for assessing readability and comprehensibility. Consequently, this particular aspect has not been addressed in the present study.

In conclusion, industrial/commercial profit organizations were the most prevalent producers of web pages, but the information provided by these entities was of the lowest quality. Most short videos were created by healthcare providers, but the overall quality of these videos was found to be inadequate. In general, the quality of both web pages and short videos is inadequate. However, the quality of web pages was superior to that of short videos. In terms of completeness and accuracy, both the web page and the video exhibit significant deficiencies that are cause for concern. Considering the growing prevalence of the Internet, it is imperative to reinforce the regulatory framework and quality control measures pertaining to content on these platforms. It would be prudent for individuals to exercise caution when accessing healthcare management information on the Internet.

## Data Availability

The raw data supporting the conclusions of this article will be made available by the authors, without undue reservation.
