# Supplementary material for: Search engines and short video apps as sources of information on acute pancreatitis in China: quality assessment and content assessment
Source: Front Public Health. 2025 Jun 4;13:1578076. doi: 10.3389/fpubh.2025.1578076 (PMC12174125; doi:10.3389/fpubh.2025.1578076)
Supplement: Supplementary file 1 [file Data_Sheet_1.DOCX]

| Table1: DISCERN SCORE |
| --- |
| 1. Are the aims clear? |
| 1. Does it achieve its aims? |
| 1. Is it relevant? |
| 1. Is it clear what sources of information were used to compile the publication (other than the author or producer)? |
| 1. Is it clear when the information used or reported in the publication was produced? |
| 1. Is it balanced and unbiased? |
| 1. Does it provide details of additional sources of support and information? |
| 1. Does it refer to areas of uncertainty? |
| Section2: HOW GOOD IS THE QUALITY OF INFORMATION ON TREATMENT CHOICES? |
| 1. Does it describe how each treatment works? |
| 1. Does it describe the benefits of each treatment? |
| 1. Does it describe the risks of each treatment? |
| 1. Does it describe what would happen if no treatment were used? |
| 1. Does it describe how the treatment choices aVect overall quality of life? |
| 1. Is it clear that there may be more than one possible treatment choice? |
| 1. Does it provide support for shared decision-making? |
| Section 3: OVERALL RATING OF THE PUBLICATION |
| 1. Based on the answers to all of the above questions, rate the overall quality of the publication as a source of information about treatment choices: |

Table2: The JAMA Benchmark Criteria

| Authorship | Authors and contributors, their affiliations, and relevant credentials should be provided. |
| --- | --- |
| Attribution | References and sources for all content should be listed clearly, and all relevant copyright information noted. |
| Disclosure | Web site "ownership" should be prominently and fully disclosed, as should any sponsorship, advertising, underwriting, commercial funding arrangements or support, or potential conflicts of interest. This includes arrangements in which links to other sites are posted because of financial considerations. Similar standards should hold in discussion forums. |
| Currency | Dates that content was posted and updated should be indicated. |

Table3: GQS（Global quality Score）

| 1 | Poor quality,poor flow of the site,most information missing,not at all useful for patients |
| --- | --- |
| 2 | Generally poor quality and poor flow, some information listed but many important topics missing, of very limited use to patients |
| 3 | Moderate quality, suboptimal flow, some important information is adequately discussed but others poorly discussed, somewhat useful for patient |
| 4 | Good quality and generally good flow, most of the relevant information is listed, |
| 5 | Excellent quality and excellent flow, very useful for patients |

Table4: Content

| **Etiology** |
| --- |
| 1. Gallstones (21%-33%) |
| 1. Alcohol (16%-27%) |
| 1. Triglyceridemic (2%-5%) |
| 1. Medical origin (ERCP/EUS) |
| 1. Hypercalcemia |
| 1. Infection |
| **Clinical Presentation** |
| 1. Abdominal pain |
| 1. Nausea |
| 1. Vomiting |
| 1. Fever |
| **Diagnostic Criteria** |
| The Atlanta Classification (RAC) requires 2 of the following 3 criteria: (1) abdominal pain suggestive of pancreatitis, (2) serum amylase and/or lipase greater than 3 times the upper limit of normal, and (3) cross-sectional imaging (CT or MRI) findings consistent with acute pancreatitis. |
| Disease Severity |
| 1. Mild acute pancreatitis: no local complications or organ failure. |
| 1. Moderate severe acute pancreatitis: transient organ failure (recovery within 48 hours) and/or localized complications. |
| 1. Severe acute pancreatitis: persistent organ failure for more than 48 hours with or without local complications. |
| **Risk Stratification** |
| 1. APACHE II |
| 1. BISAP |
| 1. Ranson |
| **Management** |
| 1. Analgesic |
| 1. Fluid resuscitation: lactated Ringer's solution |
| **Relapse Prevention** |
| 1. Cholecystectomy |
| 1. Alcohol cessation strategies |
| 1. Low Fat Diet |
| 1. Weight loss |

Tablel5: Guide Score

| 1. Pancreatography-enhanced computed tomography (CECT) and/or magnetic resonance imaging (MRI) should be reserved for patients whose diagnosis is unclear or who are not clinically better within 48 - 72 hours of admission (highly recommended, low quality of evidence). |
| --- |
| 1. Transabdominal ultrasound should be performed in all patients with acute pancreatitis (highly recommended, low quality of evidence). |
| 1. Hemodynamic status should be assessed immediately upon presentation and resuscitative measures initiated as needed (strong recommendation, moderate quality of evidence). |
| 1. Patients with organ failure should be admitted to an intensive care unit or intermediate care facility whenever possible (strong recommendation, low quality of evidence). |
| 1. Aggressive hydration, defined as 250-500 mL per hour of isotonic crystalloid solution, should be provided to all patients unless cardiovascular and/or renal comorbidities are present. Early aggressive intravenous hydration is most beneficial in the first 12 - 24 hours and may be of little benefit thereafter (highly recommended, moderate quality of evidence). |
| 1. ERCP should be performed within 24 hours of admission in patients with acute pancreatitis combined with acute cholangitis (strongly recommended, moderate quality of evidence). |
| 1. ERCP is not required in most patients with gallstone pancreatitis who lack laboratory or clinical evidence of persistent biliary obstruction (strong recommendation, low quality of evidence). |
| 1. Antibiotic therapy should be given for extrapancreatic infections such as cholangitis, catheter-acquired infections, bacteremia, urinary tract infections, and pneumonia (strong recommendation, high quality of evidence). |
| 1. Routine use of prophylactic antibiotics in patients with severe acute pancreatitis is not recommended (strong recommendation, moderate quality of evidence). |
| 1. Antibiotics are not recommended in patients with aseptic necrosis to prevent the development of infectious necrosis (strong recommendation, moderate quality of evidence). |
| 1. Enteral nutrition is recommended in severe AP to prevent infectious complications. Parenteral nutrition should be avoided unless the enteral route is unavailable, not tolerated, or does not meet caloric needs (strong recommendation, high quality of evidence). |
| 1. Nasogastric administration and nasojejunal administration of enteral feedings appear to be equivalent in terms of effectiveness and safety (strong recommendation, moderate quality of evidence). |
| 1. In patients with mild AP who are found to have gallstones in the gallbladder, cholecystectomy should be performed before discharge to prevent recurrence of AP (strong recommendation, moderate quality of evidence). |
| 1. In patients with necrotizing biliary AP, cholecystectomy should be delayed until active inflammation has subsided and effusion has subsided or stabilized to prevent infection (strong recommendation, moderate quality of evidence). |
| 1. For patients with acute alcoholic pancreatitis, a brief alcohol intervention during hospitalization is recommended. (strong recommendation) |

Table6: Percentage of short videos scored for content.

| Classification | Item | 0 | 1 |
| --- | --- | --- | --- |
| Etiology | Gallstones (21%-33%) | 56.92% | 43.08% |
|  | Alcohol (16%-27%) | 58.46% | 41.54% |
|  | Triglyceridemic (2%-5%) | 50.77% | 49.23% |
|  | Medical Origin (ERCP/EUS) | 87.69% | 12.31% |
|  | Hypercalcemia | 92.31% | 7.69% |
|  | Infection | 89.23% | 10.77% |
| Clinical Presentation | Abdominal Pain | 56.92% | 43.08% |
|  | Nausea | 81.54% | 18.46% |
|  | Vomiting | 80.00% | 20.00% |
|  | Fever | 90.77% | 9.23% |
| Diagnostic Criteria | The Atlanta Classification (RAC) Requires 2 Of The Following 3 Criteria: (1) Abdominal Pain Suggestive Of Pancreatitis, (2) Serum Amylase And/Or Lipase Greater Than 3 Times The Upper Limit Of Normal, And (3) Cross-Sectional Imaging (CT Or MRI) Findings Consistent With Acute Pancreatitis. | 92.31% | 6.15% |
| Disease Severity | Mild Acute Pancreatitis: No Local Complications Or Organ Failure. | 86.15% | 13.85% |
|  | Moderate Severe Acute Pancreatitis: Transient Organ Failure (Recovery Within 48 Hours) And/Or Localized Complications. | 86.15% | 13.85% |
|  | Severe Acute Pancreatitis: Persistent Organ Failure for More Than 48 Hours With Or Without Local Complications. | 86.15% | 13.85% |
| Risk Stratification | APACHE II | 98.46% | 1.54% |
|  | BISAP | 98.46% | 1.54% |
|  | Ranson | 98.46% | 1.54% |
| Management | Analgesic | 92.31% | 7.69% |
|  | Fluid Resuscitation: Lactated Ringer's Solution | 96.92% | 3.08% |
|  | Nutrition: Enteral Nutrition | 98.46% | 1.54% |
|  | Lipid Management | 96.92% | 3.08% |
| Relapse Prevention | Cholecystectomy | 96.92% | 3.08% |
|  | Alcohol Cessation Strategies | 87.69% | 12.31% |
|  | Low Fat Diet | 80.00% | 20.00% |
|  | Weight Loss | 92.31% | 7.69% |

Table7: Percentage of web pages scored for content.

| Classification | Item | 0 | 1 |
| --- | --- | --- | --- |
| Etiology | Gallstones (21%-33%) | 26.53% | 73.47% |
|  | Alcohol (16%-27%) | 32.65% | 67.35% |
|  | Triglyceridemic (2%-5%) | 32.65% | 67.35% |
|  | Medical origin (ERCP/EUS) | 51.02% | 48.98% |
|  | Hypercalcemia | 53.06% | 46.94% |
|  | Infection | 55.10% | 44.90% |
| Clinical Presentation | Abdominal pain | 44.90% | 55.10% |
|  | Nausea | 28.57% | 71.43% |
|  | Vomiting | 38.78% | 61.22% |
|  | Fever | 42.86% | 57.14% |
| Diagnostic Criteria | The Atlanta Classification (RAC) requires 2 of the following 3 criteria: (1) abdominal pain suggestive of pancreatitis, (2) serum amylase and/or lipase greater than 3 times the upper limit of normal, and (3) cross-sectional imaging (CT or MRI) findings consistent with acute pancreatitis. | 77.55% | 22.45% |
| Disease Severity | Mild acute pancreatitis: no local complications or organ failure. | 73.47% | 26.53% |
|  | Moderate severe acute pancreatitis: transient organ failure (recovery within 48 hours) and/or localized complications. | 73.47% | 26.53% |
|  | Severe acute pancreatitis: persistent organ failure for more than 48 hours with or without local complications. | 73.47% | 26.53% |
| Risk Stratification | APACHE II | 85.71% | 14.29% |
|  | BISAP | 100.00% | 0.00% |
|  | Ranson | 100.00% | 0.00% |
| Management | Analgesic | 69.39% | 30.61% |
|  | Fluid resuscitation: lactated Ringer's solution | 57.14% | 42.86% |
|  | Nutrition: Enteral Nutrition | 85.71% | 14.29% |
|  | Lipid Management | 57.14% | 42.86% |
| Relapse Prevention | Cholecystectomy | 77.55% | 22.45% |
|  | Alcohol cessation strategies | 77.55% | 22.45% |
|  | Low Fat Diet | 79.59% | 20.41% |
|  | Weight loss | 77.55% | 22.45% |
